# Molecular Vibrations in Chiral Europium Complexes Revealed by Near‐Infrared Raman Optical Activity

**DOI:** 10.1002/advs.202305521

**Published:** 2023-11-20

**Authors:** Tao Wu, Petr Bouř, Tomotsumi Fujisawa, Masashi Unno

**Affiliations:** ^1^ Institute of Organic Chemistry and Biochemistry Czech Academy of Sciences Flemingovo náměstí 2 Prague 166 10 Czech Republic; ^2^ Department of Chemistry and Applied Chemistry Faculty of Science and Engineering Saga University Saga 840‐8502 Japan

**Keywords:** chiral lanthanide complexes, circularly polarized luminescence, density functional theory, Raman optical activity, spectra simulations

## Abstract

Raman optical activity (ROA) is commonly measured with green light (532 nm) excitation. At this wavelength, however, Raman scattering of europium complexes is masked by circularly polarized luminescence (CPL). This can be avoided using near‐infrared (near‐IR, 785 nm) laser excitation, as demonstrated here by Raman and ROA spectra of three chiral europium complexes derived from camphor. Since luminescence is strongly suppressed, many vibrational bands can be detected. They carry a wealth of structural information about the ligand and the metal core, and can be interpreted based on density functional theory (DFT) simulations of the spectra. For example, jointly with ROA experimental data, the simulations make it possible to determine absolute configuration of chiral lanthanide compounds in solution.

## Graphical abstract

Raman optical activity using near‐IR laser excitation with the aid of density functional theory calculations provides a reliable approach for the determination of absolute configuration of chiral lanthanide compounds in solution.

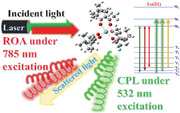

## 1 Introduction

Luminescent lanthanide(III) complexes have generated interest in biomedical analyses and imaging, owing to their characteristic electronic structure and sensitivity to the environment.^[^ ^1^ ^]^ A detailed insight into their geometry and internal energy transfer is instrumental for rational design and characterization of luminescent probes and other functional materials. Exploring specific interactions of chiral molecules with left‐ and right‐circularly polarized light, chiroptical spectroscopies are indispensable in stereochemical analyses of the lanthanide complexes.^[^ ^2^ ^]^

Among chiroptical methods, electronic circular dichroism (ECD) is probably the most frequently used technique in structural studies of chiral molecules, and is capable of sensing the electronic properties of the ground state. Typically, dissymmetry factor (*g*_ECD_) is ≈10^−3^ for ligand chromophores in lanthanide complexes with normal concentration. But it can vary in a broad interval and become close to one for some *f*–*f* transitions. The factor is defined as *g*_ECD_ = 2(*ε*_L_ − *ε*_R_)/(*ε*_L_ + *ε*_R_), where *ε*_L_ and *ε*_R_ are molar extinction coefficients of the left‐ and right‐circularly polarized light.^[^ ^3^ ^]^

CPL is usually more suitable than ECD to study the lanthanide(III) complexes.^[^ ^4^ ^]^ It probes the excited electronic states, and its dissymmetry factor (*g*_lum_) is sometimes associated with the *f–f* transitions and thus often greater than *g*_ECD_ associated with ligand‐centered transitions. For luminescence, the factor is defined as *g*_lum_ = 2(*I*_L_ − *I*_R_)/(*I*_L_ + *I*_R_), where *I*_L_ and *I*_R_ are the intensities of emitted left‐ and right‐circularly polarized light. For a camphor‐derived diketonate Eu(III) complex with cesium, an extremely high *g*_lum_ value of 1.38 was reported.^[^ ^5^ ^]^ The drawback of the electronic methods is that they can reveal only a limited number of transitions, and their interpretation through computations is problematic. In general, the ECD and CPL spectroscopies are complementary; typically some *f*–*f* transitions not observable in one may be measurable in the other.

Vibrational chiroptical spectroscopy is capable of revealing more and better resolved spectral bands, and senses the structure more locally than ECD or CPL.^[^ ^6^ ^]^ For example, vibrational circular dichroism (VCD) spectroscopy is sensitive not only to the absolute configuration but also to the secondary structure of proteins and nucleic acids.^[^ ^7^ ^]^ VCD of metal complexes including lanthanides has been reported as well.^[^ ^2^ ^]^ For example, VCD spectra of tetrakis((+)−3‐heptafluorobutylyrylcamphorato) Ln(III) complexes can discriminate between encapsulated alkali metal ions.^[^ ^8^ ^]^ VCD spectra of Cs(I) ion and Ln(III) complexes depend on the state parity and orbital angular momentum.^[^ ^9^ ^]^ Lanthanide ions sometimes amplify the VCD intensities of the ligand vibrations.^[^ ^10^ ^]^ An intensity enhancement has been documented for an α‐pinene‐derived bipyridine‐Eu(III) complex,^[^ ^11^ ^]^ and is explicable by the beyond‐Born‐Oppenheimer state mixing,^[^ ^12^ ^]^ when the electronic and vibrational energy levels in the complex are comparable.^[^ ^13^ ^]^ Despite of the general knowledge, current theory is not able to provide a routine support for VCD of chiral lanthanide complexes.^[^ ^9^, ^14^ ^]^

Vibrational ROA spectroscopy is also employed to study metal complexes, even though less frequently than the other chiroptical techniques.^[^ ^2^, ^15^ ^]^ Here, an important measure of chirality is the normalized circular intensity difference [*CID =* (*I*_R_ − *I*_L_)/(*I*_R_ + *I*_L_)] the ratio of ROA and Raman intensities, which is an equivalent of the dissymmetry factor in ECD, VCD and CPL. Typically, *CID* <10^−3^. The energy of a commercially available green (532 nm) laser is close to that of the ^7^F_0_ → ^5^D_1_ and ^7^F_1_ → ^5^D_1_ electronic transitions of the Eu (III) ion. This is in principle suitable to invoke resonance ROA^[^ ^16^ ^]^ and some authors thus suggested that the lanthanide element could produce an intense and easily detectable signal in both Raman^[^ ^17^ ^]^ and ROA^[^ ^18^ ^]^ spectra. The so‐called “induced” resonance ROA spectroscopy was employed for chiral analyses of optically active alcohols and ketones in the presence of an achiral europium (III) complex.^[^ ^19^ ^]^ The CID value of 10^−2^ was higher than in most organic molecules and biomolecules.^[^ ^7a^ ^]^ A similar CID value was also reported for enantiomers of α‐pinene‐derived bipyridine‐Eu(III) complex.^[^ ^11^ ^]^ Today we know that the signal mostly comes from lanthanide CPL, not ROA, but this phenomenon greatly facilitates measurements of the chiral signal as lower sample concentrations and/or shorter measurement times are feasible.

In fact, Raman and luminescence bands can be easily distinguished based on measurements with different excitation wavelengths or degrees of circularity.^[^ ^20^ ^]^ CPL is sometimes induced in the symmetrical Eu(III) ion by the chiral milieu. Note that both ROA and CPL measure differences in scattering of the right‐ and left‐circularly polarized light, they can be obtained on the same spectrometer, and a spectrum can contain both signals.^[^ ^7^, ^21^ ^]^ The definitions are therefore somewhat unfortunate, but the experimental data can be easily converted (ROA = −CPL and *g*_lum_ = −2*CID*). ROA itself has been used to study small organic molecules, metal complexes and biomolecules.^[^ ^2^, ^22^ ^]^

Recently, we have demonstrated that not only CPL, but also ECD can affect ROA data.^[^ ^23^ ^]^ For example, a strong induced “false ROA” signal of the solvent may appear.^[^ ^23^, ^24^ ^]^ Fortunately, the absorption and ECD of lanthanide complexes in the region of interest (≈530–700 nm) is extremely weak,^[^ ^25^ ^]^ and in cases presented here it is expected to have a negligible effect on ROA spectra.

Measurement of CPL spectra on a commercial ROA spectrometer employing green laser excitation is very convenient for europium(III) compounds.^[^ ^20^, ^26^ ^]^ The metal exhibits sharp CPL lines (especially for the ^5^D_0_ →^7^F_1_ transition) that fit into the instrumental spectral window. On the other hand, the genuine ROA signal cannot be measured due to the strong luminescence. Other Eu(III) luminescence bands such as those due to the ^5^D_0_ → ^7^F_4_ (680–710 nm) or ^5^D_0_ → ^7^F_6_ (810–840 nm) transitions lie outside of spectrometer's sensitivity range.^[^ ^27^ ^]^

**Scheme 1:**
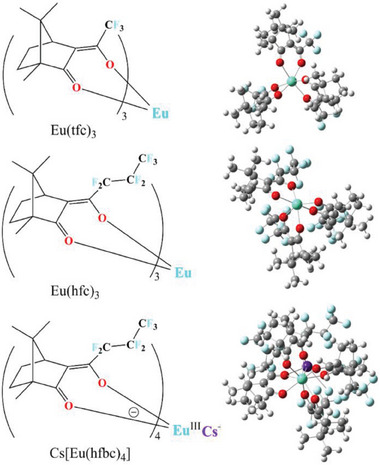
Chemical structures and optimized geometries (DFT level, Gaussian program^[^ ^35^ ^]^) of the studied Eu(III) complexes.

Using near‐IR (785 nm) laser excitation, however, we could eliminate the CPL bands, and reveal the genuine vibrational ROA signal of three chiral europium complexes (**Scheme** **1**): europium tris[3‐(trifluoromethylhydroxymethylene)‐(+)‐camphorate] [Eu(tfc)_3_], europium tris[3‐(heptafluoropropylhydroxymethylene)‐(+)‐camphorate] [Eu(hfc)_3_], and cesium tetrakis(3‐heptafluorobutylryl‐(+)‐camphorate) europium complex, Cs[Eu(hfbc)_4_]. These complexes have already been used in various spectroscopic applications; Eu(tfc)_3_ and Eu(hfc)_3_ serve as chiral shift reagents in NMR spectroscopy, while Cs[Eu(hfbc)_4_] is used as a CPL calibration standard, owing to its huge dissymmetry factor.

## 2 Results and Discussion

Measurements of the Eu(tfc)_3_ complex with the green laser (532 nm) have been described elsewhere.^[^ ^18^ ^]^ Because of the proportionality of ROA signal to the fifth power of inverse wavelength (≈λ^−5^),^[^ ^7^, ^21^ ^]^ at the near‐IR (785 nm) excitation the spectra accumulation is more difficult. While Raman bands of the Eu(tfc)_3_ complex could be detected within minutes, several days were needed to obtain ROA. Fortunately, the complex was quite stable in chloroform and Raman and ROA spectral patterns did not change even after 140 h.

**Figure 1:**
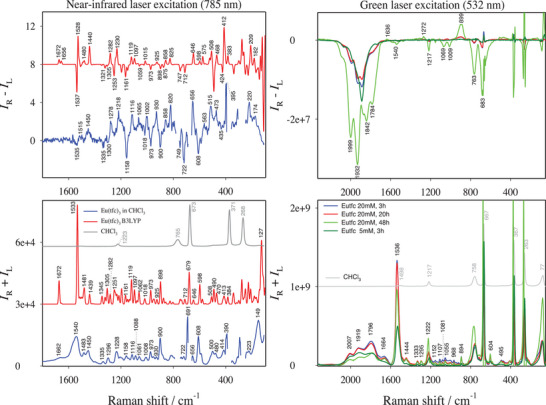
Raman (*I*_R_ + *I*_L_) and ROA (*I*_R_ − *I*_L_) spectra of the Eu(tfc)_3_ complex. Grey traces denote neat solvent (CHCl_3_). (Left) 20 mm solution in CHCl_3_ at near‐IR (785 nm, blue for experimental spectra and red for simulated ones by DFT calculation at B3LYP level). Artifacts due to solvent signal saturation have been removed (gaps). (Right) 20 mm and 5 mm solution in CHCl_3_ at green‐light (532 nm) excitation.

As shown in **Figure** **1** (left), Raman and ROA spectra of the Eu(tfc)_3_ complex at near‐IR excitation feature many bands and thus convey a wealth of structural information. Many spectral features are already discernible in the spectra of the ligand alone (Figure S1, Supporting Information). Most of the bands could be assigned on the basis of DFT simulation. Additional simulations are shown in Figure S2 (Supporting Information). The ^5^D_0_ → ^7^F_6_ transition (810‐840 nm),^[^ ^27^ ^]^ which is occasionally measurable, was not detected either by total luminescence (Raman trace) or by CPL (ROA trace), although the transition located within the near‐IR ROA spectral window (≈790–960 nm).

The main vibrational bands are summarized in **Table** **1**. The C═O stretching yields a very weak Raman band at 1662 cm^−1^. Predictably, weak ROA bands were not detected. Both Raman and ROA bands at 1450 cm^−1^ of the C═O stretch mode are only moderately strong, and are accompanied by C─H bending signal of the bicyclic skeleton. The broad Raman band at 1540 cm^−1^ is mainly due to C═C stretching of the diketonate O─C─C═C─C═O group^[^ ^28^ ^]^ yielding a weak bisignate ROA couplet (−/+) at 1535/1515 cm^−1^. The weak Raman band at 1158 cm^−1^ is due to C─H bending and C─CH_3_ stretching of the bicyclic skeleton, and corresponds to a relatively strong negative ROA band at 1158 cm^−1^. The CH_3_ rocking signal is clearly visible in both Raman and ROA at 973 cm^−1^.

**Table 1:** Selected Raman bands of the three europium diketonate complexes.

Raman shift [cm^−1^] measured (calculated) in Eu(III) complexes			Band assignment
Eu(tfc)_3_	Eu(hfc)_3_	Cs[Eu(hfbc)_4_]	
1662 (1672)	1659 (1682, 1663)	1686, 1659 (1735, 1704)	C═O stretching
1540 (1533)	1538 (1544)	1550, 1533 (1598, 1527)	C═C stretching
1480 (1481)	1480 (1493)	1483 (1503)	CH_3_ scissoring
1450 (1439)	1450 (1473)	1448 (1484)	C─H bending and C═O stretching
1158 (1161)	1160 (1166)	1162 (1177)	C─H bending and C─CH_3_ stretching
973 (973)	975 (966)	975 (974)	CH_3_ rocking

At the green‐light excitation (Figure 1, right), we see a very different picture. Luminescence/CPL bands of the Eu(III) ^5^D_0_ → ^7^F_1_ transition dominate Raman/ROA spectra within ≈1600–2050 cm^−1^ interval. As mentioned above, these were previously incorrectly identified as resonance ROA bands.^[^ ^18^ ^]^ Their combined intensity is comparable to that of Raman bands within 490–1500 cm^−1^ , and most of the latter can also be detected under near‐IR excitation. However, at green‐light excitation, the “ROA” trace (top panel) is dominated by strong CPL and corresponding *g*_lum_ (= −2*CID*) is as high as ≈10^−2^. The vibrational ROA is not simultaneously detectable as *CID* is low, ≈10^−4^. Interestingly, the CPL spectra are significantly modulated by concentration (5 mm vs 20 mm), which suggests some longer‐range or complex‐complex interactions.^[^ ^29^ ^]^ In addition, CPL‐ECD interference may contribute to measured ROA, especially that of *f*–*f* transitions involving ground state ^7^F_0_ at higher concentration. Long acquisition times also bring about some spectral changes, e.g. CPL bands of the Eu(III) ^5^D_0_ → ^7^F_1_ transition within ≈1600–2050 cm^−1^ and ^5^D_1_ → ^7^F_2_ transition within ≈700–1100 cm^−1^ are enhanced significantly. This indicates that the Eu(tfc)_3_ complex in chloroform may partially decompose.

**Figure 2:**
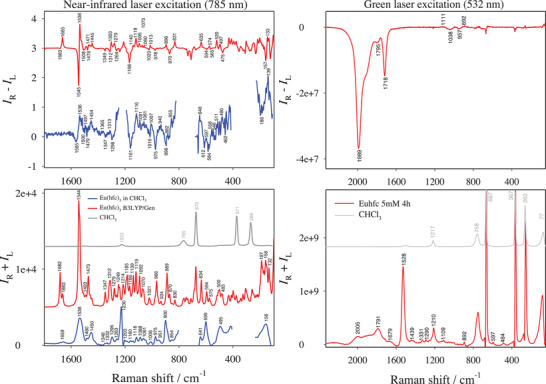
Raman (*I*_R_ + *I*_L_) and ROA (*I*_R_ – *I*_L_) spectra of the Eu(hfc)_3_ complex. Grey traces denote the solvent. (Left) 20 mm solution in CHCl_3_ at 785 nm excitation (blue for experimental spectra and red for simulated ones by DFT calculation at B3LYP level). Artifacts due to solvent signal saturation have been removed (gaps). (Right) 5 mm in CHCl_3_ at 532 nm excitation.

As shown in **Figure** **2** (left), the Eu(hfc)_3_ complex behaves slightly differently than Eu(tfc)_3_. Artifacts in the ROA bands within the 810–618 cm^−1^ and 410–230 cm^−1^ regions are removed for clarity; they are due to detector saturation by Raman bands of chloroform (CHCl_3_) vibrations. Raman bands were apparent within a few minutes under near‐IR excitation, and about 5 days were required for ROA. Raman and some true molecular ROA bands are clearly visible under near‐IR excitation, mainly due to the 3‐(heptafluoropropylhydroxymethylene)‐(+)‐camphorate ligand (Figure S3, Supporting Information). Most of the observed bands could be reproduced by DFT calculations. Again, the C═O stretching yields a very weak Raman band at 1659 cm^−1^ and no visible ROA signal. The Raman band at 1450 cm^−1^ of C═O stretching accompanied by C─H bending is of moderate intensity, and an accompanying ROA band at 1454 cm^−1^ is rather weak. The broad Raman band at 1538 cm^−1^ is attributed to C═C stretching of the diketonate O─C─C═C─C═O group^[^ ^28^ ^]^ (Table 1), with a ROA couplet (−/+) at 1565/1536 cm^−1^; and a weak Raman band at 1160 cm^−1^ to C─H bending and C─CH_3_ stretching. A strong negative ROA band at 1161 cm^−1^ is visible despite the presence of a strong solvent Raman signal nearby. The CH_3_ rocking mode generates Raman and ROA bands at 975 cm^−1^.

At green‐light excitation (Figure 2, right), Eu(III) luminescence and CPL bands are again visible. The CPL signal is consistent with a previously reported measurement in deuterated acetone using a conventional CPL spectrometer.^[^ ^30^ ^]^

**Figure 3:**
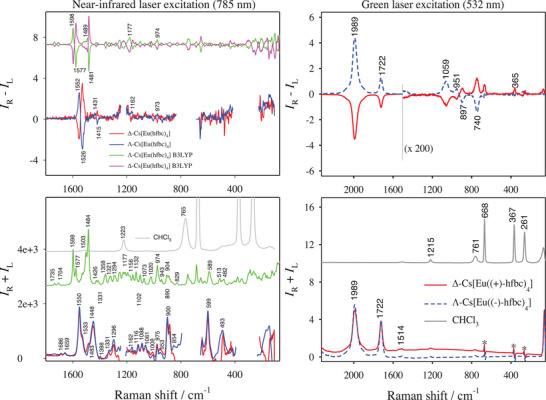
Raman (*I*_R_ + *I*_L_) and ROA (*I*_R_ – *I*_L_) spectra of Δ /Λ‐Cs[Eu(hfbc)_4_] in CHCl_3_. Grey traces denote the solvent (CHCl_3_) alone. (Left) 10 mm solution at near‐IR (785 nm) excitation (red/blue for experimental spectra of Δ /Λ‐Cs[Eu(hfbc)_4_] and pink/green for simulated ones by DFT calculation at B3LYP level). Artifacts due to the solvent signal saturation have been removed (gaps). (Right) 2 mm at green‐light excitation (Reproduced with permission^[^ ^20^ ^]^ Copyright 2015, John Wiley and Sons). Top trace has been expanded below 1500 cm^−1^. Asterisks (bottom trace) denote artifacts due to solvent signal subtraction.

Finally, spectra of the Cs[Eu(hfbc)_4_] complex are shown in **Figure** **3**. This compound is not very soluble. This was not a problem for Raman spectrum, but ROA measurement took up to (≈6 days) at near‐IR excitation.

Some Raman bands can be found even in spectra of the 3‐(heptafluoropropylhydroxymethylene)‐(+/−)‐camphorate ligand (Figure S3, Supporting Information). Similarly as in Eu(hfc)_3_, most Raman bands not masked by the solvent can be also reproduced by DFT (Figure 3, left). ROA bands are often hidden in the noise, but the visible ones are predicted with the right sign. The C═O stretching yields two weak Raman bands at 1686 and 1659 cm^−1^. As in the other two complexes, the experimental ROA signal is not detectable. However, there is a broad Raman band at 1448 cm^−1^ assigned to C═O stretching, and it is accompanied by C─H bending and a corresponding ROA couplet (+/−) at 1431/1415 cm^−1^. A narrower Raman band at 1550 cm^−1^ and its side peak at 1533 cm^−1^ are assigned to C═C stretching in the diketonate O─C─C═C─C═O group^[^ ^28^ ^]^ (Table 1). It also yields a very strong ROA couplet (+/−) at 1552/1526 cm^−1^. The weak Raman band at 1162 cm^−1^ is mainly due to C‐H bending and C─CH_3_ stretching in the bicyclic skeleton, and the weak positive ROA band at 1162 cm^−1^ is still discernible despite of the strong solvent signal. The CH_3_ rocking mode can be easily identified in both ROA/Raman bands at 973/975 cm^−1^.

At the green‐light excitation (Figure 3, right) and unlike in the other two complexes, luminescence is so strong that Raman bands of ligand are almost invisible unless rescaled. Obviously, the extremely strong CPL featuring *g_lum_* close to unity prevents any vibrational ROA bands from being easily detected.^[^ ^20^ ^]^ The luminescence bands are mostly attributed to the ^5^D_0_ → ^7^F_1_ Eu(III) transition.

**Table** **2** summarizes the data obtained on all three complexes. It is interesting that maximal *CID* values *CID*_max_ obtained at 532 and 785 nm excitations follow the same trend, even though the chiral phenomena (ROA vs CPL) are very different. *CID* is in both cases largest for the Cs[Eu(hfbc)_4_] complex and smallest for the Eu(tfc)_3_ complex.

**Table 2:** ROA/Raman spectra of lanthanide complexes in brief, at two different wavelength excitations. *I*_R_/*I*_L_, right/left circularly polarized light.

Complex	785 nm				532 nm	
	*I_R_‐I_L_*	*I_R_+I_L_*	*CID*_max_	*I_R_‐I_L_*	*I_R_+I_L_*	*CID*_max_
Eu(tfc)_3_	ROA visible	Raman bands visible	≈4 × 10^−5^	CPL only visible	Mixed TL and Raman bands of comparable intensity	≈0.015
Eu(hfc)_3_	Weaker ROA	Raman bands visible	≈10^−4^	CPL only visible	Strong TL, weak Raman	0.25
Cs[Eu(hfbc)_4_]	Weaker ROA	Raman bands visible	≈2.5 ×10^−4^	CPL only visible	Strong TL, Raman nearly invisible	0.71

Note that except for the differences in laser wavelength the two ROA instruments adopt the same scattered‐circular polarization (SCP) mode, and use a similar optical setup. Near‐IR measurement of vibrational ROA is more difficult but has many advantages over other chiroptical modalities such as ECD,^[^ ^25^, ^31^ ^]^ CPL^[^ ^32^ ^]^ and VCD.^[^ ^8^ ^]^ In particular, the luminescence is suppressed, many vibrational transitions can be detected, and the far from resonance ROA can be reliably simulated by DFT approaches.

## 3 Conclusion

We recorded Raman and ROA spectra of chiral europium complexes under the near‐IR laser excitation. This practically eliminated sample luminescence and made it possible to observe the genuine vibrational transitions. They were not detectable in more conventional Raman spectroscopy carried out using the green light excitation. At near‐IR, most of the recorded Raman and ROA bands could be reproduced by DFT calculations. On the other hand, long accumulation times were needed and relatively high noise levels still remained in the spectra. The near‐IR ROA technique as such, however, is well suited for structural studies of lanthanide complexes in solution by providing rich information on stereo‐chemistry, absolute configuration and conformation. The combination of more spectroscopic techniques and analysis of the spectra helped us to gain an insight into the molecular electronic and vibrational energy levels.

## 4 Experimental Section

### Chemicals and Spectral Measurement

The Eu(tfc)_3_ and Eu(hfc)_3_ complexes were purchased from Sigma Aldrich, and the Cs[Eu(hfbc)_4_] complex was prepared as described elsewhere.^[^ ^33^ ^]^ Backscattering SCP Raman and ROA spectra were measured on a BioTools ROA spectrometer using green laser excitation (532 nm, based in Prague), and a custom‐made spectrometer using near‐IR laser excitation (785 nm, based in Saga).^[^ ^34^ ^]^ All spectra were measured at room temperature, ROA spectra obtained at 785 nm were processed with custom‐made software to eliminate noise spikes in the spectra caused by cosmic rays, and the Raman shift was calibrated using the spectrum of neat fenchone.

### Computations

Equilibrium geometries (Scheme 1) of the three Eu(III) complexes were obtained using the Gaussian16 program^[^ ^35^ ^]^ and the B3LYP functional. For C, H, O and F atoms, the 6–311++G(d,p) basis set was employed for Eu(tfc)_3_ and Eu(hfc)_3_ complexes, and the 6–31G(d,p) basis set was employed for Cs[Eu(hfbc)_4_] complex. MWB46 and MWB28 pseudopotentials and basis sets were used for Cs in Cs[Eu(hfbc)_4_] and for Eu in all three complexes, respectively. The conductor‐like polarizable continuum solvent model (CPCM) was applied to mimic the environment (CHCl_3_).^[^ ^36^ ^]^ Although the complexes are strongly paramagnetic (with multiplicity *M* = 7), electronic excitation energies entering spectral intensities are not well‐reproduced by density functional theory (DFT). Out‐of‐resonance Raman and ROA spectra were thus generated for diamagnetic model molecules (with *M* = 1).

The reasoning of the *M* = 1 approximation for Eu(tfc)_3_ is documented in Figure S2 (Supporting Information). Here, Raman and ROA spectra are calculated with *M* = 1 and *M* = 7; and using a mixed calculation with *M* = 7 force field and geometries, but with ROA polarizability tensors^[^ ^21^ ^]^ calculated for *M* = 1. The polarizabilities were combined with the force field using the Cartesian tensor transfer techniques.^[^ ^37^ ^]^ 785 nm excitation was used throughout. The *M* = 1 and mixed cases yield almost identical spectra, which reflects the fact that geometries and force fields for *M* = 1 and *M* = 7 are very close. On the other hand, Raman and ROA intensities calculated with *M* = 7 are much higher due to a false resonance.^[^ ^7^, ^16^ ^]^ In other words, the molecule does not absorb at 785 nm, and by using *M* = 1 the unrealistic divergence of the polarizabilities was avoided, with a negligible effect on the force field.

## Conflict of Interest

The authors declare no conflict of interest.

## Supporting information

Supporting Information

## Data Availability

The data that support the findings of this study are available from the corresponding author upon reasonable request.
