# Supplementary material for: Molecular Vibrations in Chiral Europium Complexes Revealed by Near‐Infrared Raman Optical Activity
Source: Adv Sci (Weinh). 2023 Nov 20;11(1):2305521. doi: 10.1002/advs.202305521 (PMC10767399; doi:10.1002/advs.202305521)
Supplement: Supplementary file 1 — Supporting Information [file ADVS-11-2305521-s001.pdf]

ADVANCED SCIENCE
Open Access

Supporting Information

for *Adv. Sci.*, DOI 10.1002/advs.202305521

Molecular Vibrations in Chiral Europium Complexes Revealed by Near-Infrared Raman Optical Activity

Tao Wu, Petr Bouř, Tomotsumi Fujisawa and Masashi Unno**

Supporting Information

Molecular Vibrations in Chiral Europium Complexes Revealed by Near-Infrared Raman Optical Activity

Tao Wu, Petr Bouř, Tomotsumi Fujisawa, and Masashi Unno**

Contents

Figure S1. Raman and ROA spectra of 3-(trifluoroacetyl)camphor ligand enantiomers at green-light excitation

Figure S2. Raman and ROA spectra at near-IR excitation, as simulated for the $Eu(tfc)_3$ complex

Figure S3. Raman and ROA spectra of 3-(perfluorobutyryl)camphor ligand enantiomers at green-light excitation

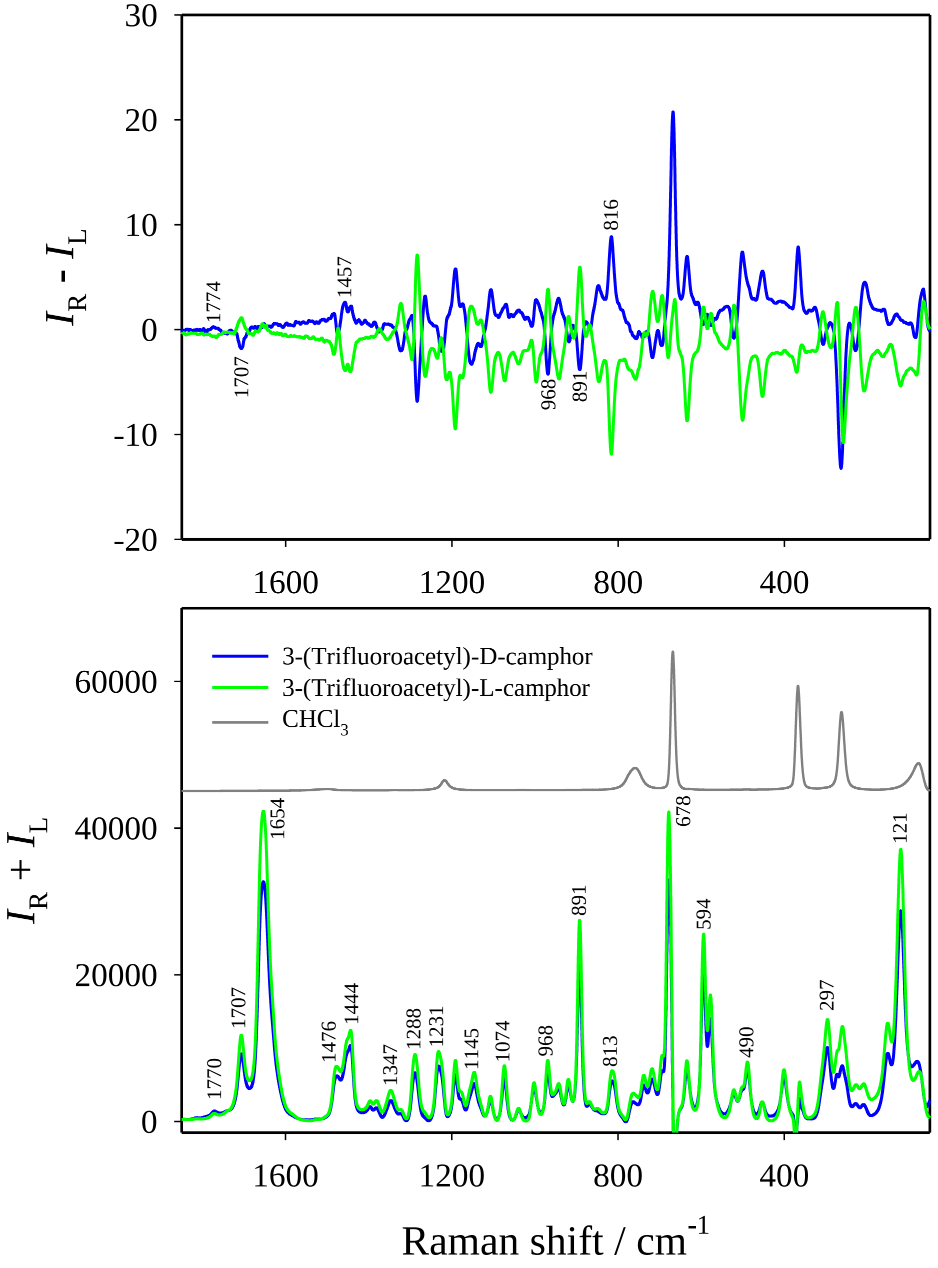

Figure S1. Raman ($I_R + I_L$) and ROA ($I_R - I_L$) spectra of the ligand 3-(trifluoroacetyl)camphor (D and L enantiomers, 1 M in $CHCl_3$) at the green-light (532 nm) excitation. Grey trace denotes the solvent ($CHCl_3$).

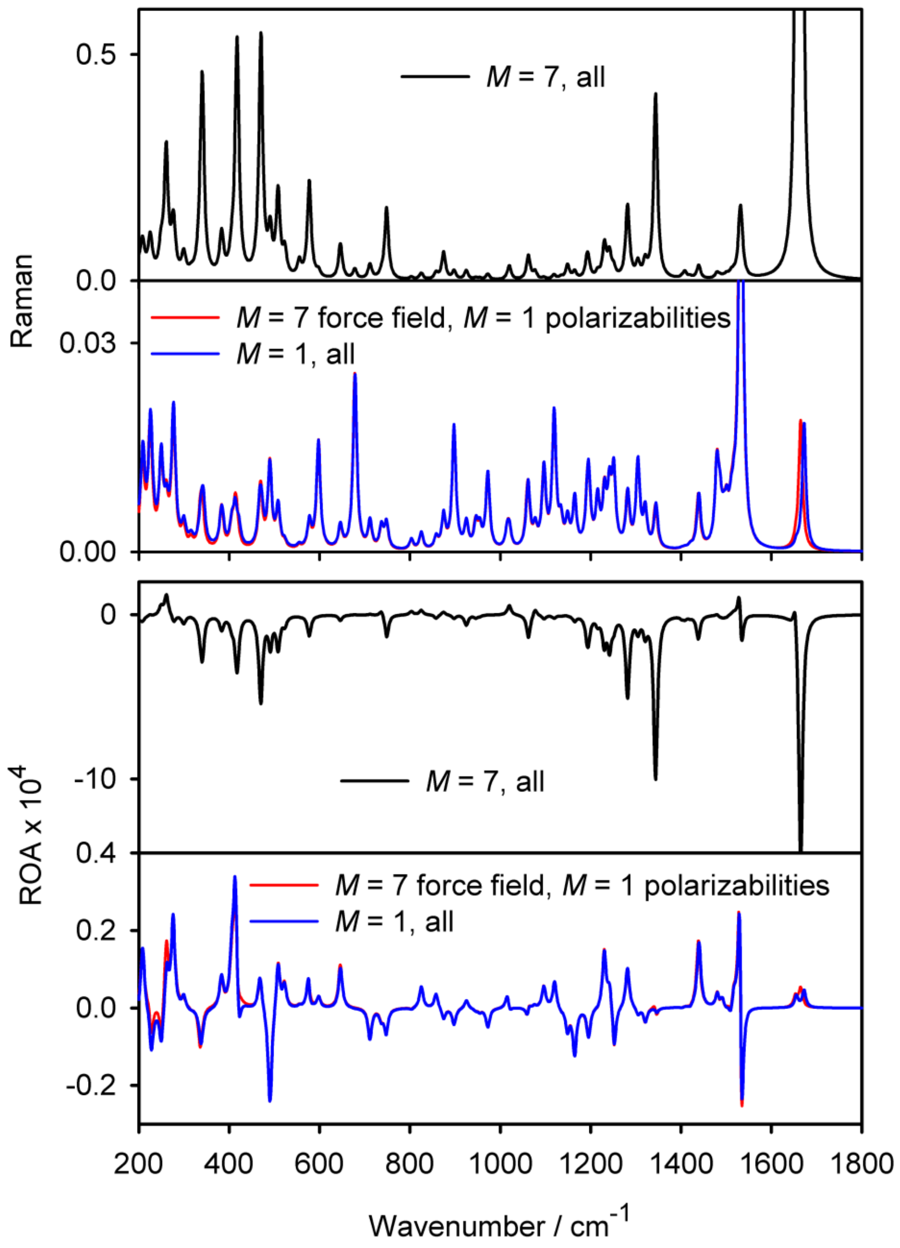

Figure S2. Raman and ROA spectra at near-IR (785 nm) excitation simulated for the $Eu(tfc)_3$ complex, with multiplicities (M) 7, 1, and a mixed calculation with force field of M = 7 and ROA polarizabilities calculated for M = 1.

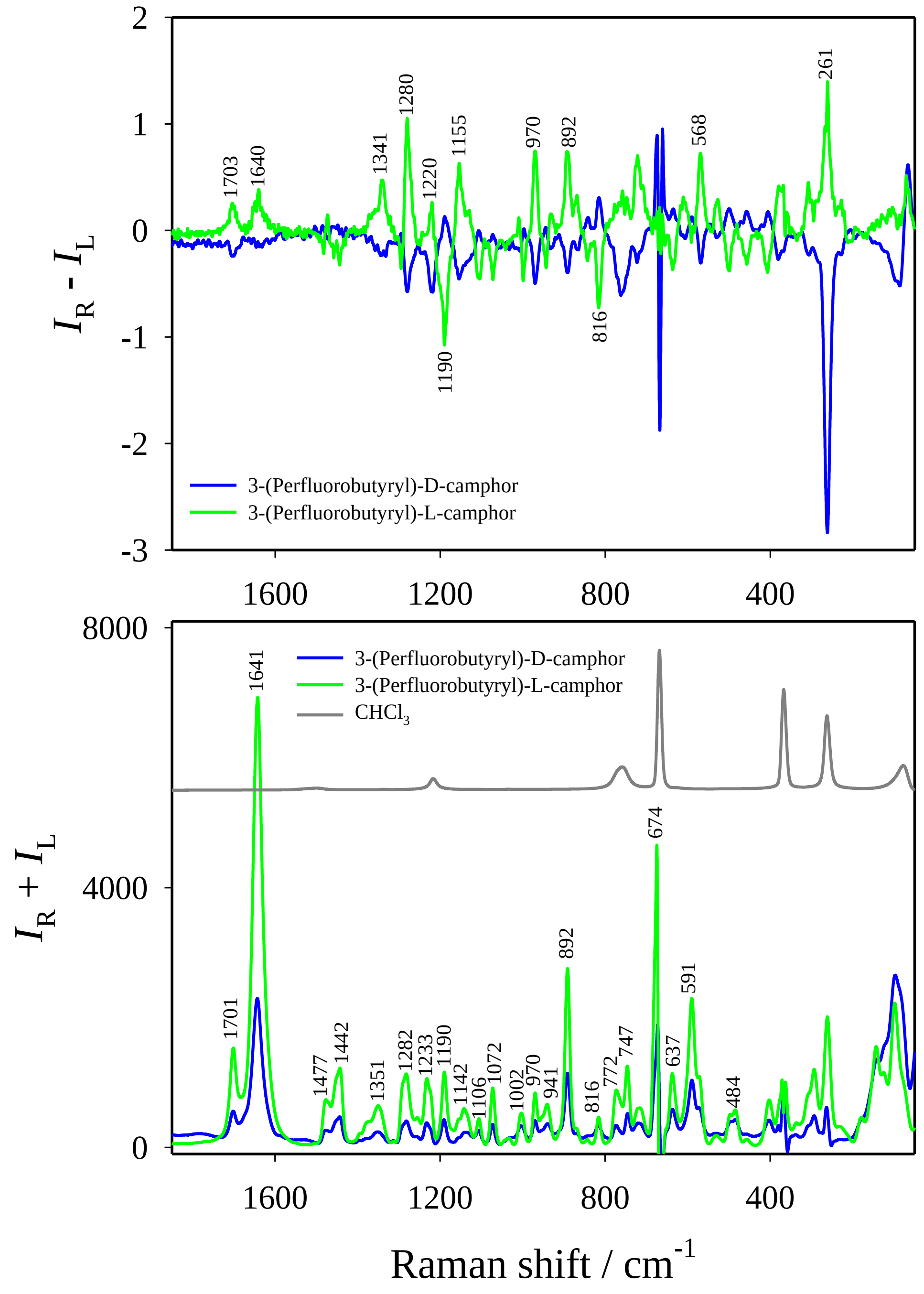

Figure S3. Raman (I_R + I_L) and ROA (I_R - I_L) spectra of the 3-(perfluorobutyryl)camphor ligand (both enantiomers, D at 0.3 M, L at 0.9 M) in $CHCl_3$ at the green-light (532 nm) excitation. Grey trace denotes the solvent ($CHCl_3$).
